# Heterogeneity Within Youth With Childhood-Onset Conduct Disorder in the ABCD Study

**DOI:** 10.3389/fpsyt.2021.701199

**Published:** 2021-07-16

**Authors:** Sarah J. Brislin, Meghan E. Martz, Lora M. Cope, Jillian E. Hardee, Alexander Weigard, Mary M. Heitzeg

**Affiliations:** ^1^Department of Psychology, Virginia Commonwealth University, Richmond, VA, United States; ^2^Department of Psychiatry, University of Michigan, Ann Arbor, MI, United States

**Keywords:** conduct disorder, latent profile analysis, CU traits, impulsivity, BIS/BAS, UPPS-P impulsive behavior scale

## Abstract

The purpose of this study was to examine if personality traits can be used to characterize subgroups of youth diagnosed with childhood-onset conduct disorder (CD). Participants were 11,552 youth from the Adolescent Brain Cognitive Development study. Data used in this report came from doi: 10.15154/1504041 (*M* age 9.92; 45.3% female, 49.6% white, 19.0% Hispanic). A subset of this sample (*n* = 365) met criteria for CD. Latent profile analyses (LPA) were performed on this subgroup (*n* = 365) to define profiles of individuals with CD based on self-report measures of impulsivity, punishment sensitivity, reward response, and callous-unemotional traits. Follow up analyses determined if these groups differed on clinically relevant variables including psychopathology, environmental risk factors, social risk factors, and neurocognitive functioning. Participants with a CD diagnosis scored significantly higher on psychological, environmental, social, and neurocognitive risk factors. The LPA revealed three unique profiles, which differed significantly on liability for broad psychopathology and domain-specific liability for externalizing psychopathology but were largely matched on environmental and social risk factors. These unique configurations provide a useful way to further parse clinically relevant subgroups within youth who meet criteria for childhood-onset CD, setting the stage for prospective longitudinal research using these latent profiles to better understand the development of youth with childhood-onset CD.

## Introduction

Conduct disorder (CD) is a set of serious emotional and behavioral problems that manifests in children and adolescents, characterized by repetitive and persistent patterns of antisocial and rule-breaking behavior. Individuals with childhood-onset CD represent a particularly high-risk group as they are more likely to have a persistent life course of CD and chronic antisocial behaviors compared to adolescent-onset subtypes (1). Childhood-onset CD is also associated with a number of unique risk factors, including comorbid psychopathology and environmental influences [for review, see (2)]. However, there is evidence that a significant number of youth with childhood-onset CD desist from antisocial behavior by early adulthood (3), suggesting that age of onset alone still designates a relatively heterogeneous group. Additional factors such as callous-unemotional (CU) traits, impulsivity, and reactivity to punishment and reward have also been shown to designate subgroups of children with CD (4–7), indicating that these traits may further clarify the heterogeneity within this high-risk group. Therefore, the current study utilized person-centered analyses in a large, diverse sample of emerging adolescents to determine if individual differences in relevant traits can define clinically meaningful subgroups of youth with childhood-onset CD.

Research on CD has focused on age of onset as one way to predict severity and recidivism. Seminal work has supported the distinction between a childhood-onset CD group, who begin showing conduct problems as early as preschool or early elementary school (8), and an adolescent-onset CD group, who begin displaying antisocial behavior with the onset of puberty (1). Research on the outcomes of these two subtypes of CD indicates that individuals with childhood-onset are more likely to continue showing antisocial behaviors into adolescence and adulthood relative to those whose behavior onsets in adolescence and are more likely to desist in adulthood (9). Childhood-onset CD is thought to reflect a more severe condition, potentially influenced by genetic predispositions, neuropsychological deficits, and exacerbated by childhood familial risk factors (e.g., parental psychopathology, family conflict) (1, 9). While the age of symptom onset is clinically relevant, the designation does not necessarily define a homogeneous group. Therefore, additional information regarding where these youth fall on relevant personality traits may inform our understanding of heterogeneity within this diagnosis.

Three relevant personality domains that may further define heterogeneity within youth diagnosed with childhood-onset CD are impulsivity, CU traits, and sensitivity to punishment and reward. There are well-established links between childhood-onset CD symptoms and greater trait impulsivity, manifesting in comorbid diagnosis of ADHD (10–14). Youth diagnosed with childhood-onset CD have higher rates of comorbid ADHD compared to those with adolescent-onset CD (15, 16) and the presence of a comorbid ADHD diagnosis predicts the persistence of antisocial behavior into adulthood (16). These findings suggest that the presence of higher levels of trait impulsivity reflect a greater risk for persistent antisocial behavior and may serve to further differentiate a high-risk group of youth.

CU traits are another personality feature that is known to increase risk for persistent antisocial behavior in youth. High levels of CU traits are exhibited by a subset of youth who meet criteria for CD (designated by the Limited Prosocial Emotions specifier in DSM-5) (17, 18). Researchers have found that youth high in CU traits have a more severe and stable trajectory of problem behavior (19–21) but also may have less anxiety, fearfulness, and sensitivity to punishment (22). Consistent with broader research on psychopathy in adults (23, 24), previous work suggests that, although impulsivity and CU traits are often correlated, these personality dimensions are dissociable and can be thought of as distinct pathways that lead to CD symptoms in youth (25–28). Research suggests that heightened levels of CU traits designate a significantly more severe group of youth, even among those who meet criteria for childhood-onset CD suggesting that level of CU traits is a clinically relevant dimension to quantify even among youth with childhood-onset CD (29, 30). Therefore, impulsive and CU traits represent two dimensions that, through different configurations, could produce different clinical outcomes for youth with a CD diagnosis.

Altered sensitivity to reward and punishment, constructs that also are particularly relevant to broader research on antisocial behavior (31, 32), have likewise been linked to CD. Specifically, research has found that youth who display CD symptoms have heightened sensitivity to reward and reduced sensitivity to punishment (33–37), with some evidence to suggest this is more pronounced in youth with childhood-onset CD (38), although inferences about the former have been somewhat inconsistent between samples and measurement modalities (39). Despite these findings, researchers have also found evidence for a subgroup of youth with CD and co-morbid anxiety disorders (i.e., psychopathology marked by hyperreactivity to threat and punishment) who demonstrate more antisocial behaviors relative to those without co-occurring internalizing psychopathology (40). Therefore, trait sensitivities to reward and punishment represent another two dimensions whereon an individual might differ but still meet criteria for CD. As these traits are closely linked to distinct neural and biological systems (31, 41, 42), determining how they co-occur could be informative for understanding the etiologic pathways to early expression of CD.

Taken together, this work demonstrates that an array of personality dimensions contribute to the early emergence of CD in youth. Latent profile analysis [LPA; (43)] uses individual data points to determine heterogeneity (i.e., different classes) among participants. While variable centered approaches such as confirmatory factor analyses use multiple variables to reduce the dimensionality of data and find homogeneity, LPA uses indicator variables to determine heterogeneity (i.e., different class membership) among participants. This analytic approach is particularly useful when there is evidence that considerable variance within each indicator variable is present, uncovering subgroups that simple group averages may fail to capture. LPA has been used to characterize heterogeneity in children with ADHD (44, 45) and in relation to other behaviors including substance use (46). The use of LPA to determine person-specific processes in CD, including the formation of meaningful subgroups through dimensional interactions, has yet to be explored. One likely reason for this limitation is that LPA often require considerably larger sample sizes than those typically present in the CD literature (47). While CD has a relatively low rate of diagnosis [lifetime prevalence of 9.5%; (48)], data from the Adolescent Brain Cognitive Development (ABCD) study are uniquely well-suited to examine subgroups within this diagnosis due to the large overall sample size and comprehensive assessments of participants and their parents.

The current study sought to first determine the trait profile of youth diagnosed with childhood-onset CD (CD+). Given that this disorder is defined by impulsive, unconstrained behavior, we hypothesized that individuals meeting criteria for childhood-onset CD would have significantly higher rates of impulsivity, lower responsivity to punishment, higher responsivity to reward, and higher levels of callous traits than the non-CD group (CD-). Given previous research detailing childhood-onset CD (6, 13) we also hypothesized that the CD+ group would have a higher proportion of males, lower levels of socioeconomic advantages (e.g., household income, parental education) and environmental enrichment (e.g., school environment, family conflict), higher rates of general psychopathology, and lower neurocognitive functioning.

Next, we predicted that different trait profiles could be defined using information regarding trait levels of impulsivity, responsivity to reward and punishment, and CU traits. To our knowledge, this is the first study to examine heterogeneity on these traits simultaneously within youth with childhood-onset CD; therefore, there is no previous research defining how these trait profiles might emerge. Based on research describing CU traits as a specifier of a high-risk subtype of CD (17, 20), we surmised that profiles would differ on this variable (i.e., defining a high-CU and low-CU groups). Similarly, previous work has defined a subgroup of youth who meet criteria for CD and also exhibit high levels of internalizing psychopathology (40). Therefore, we predicted that youth would differ on trait indices related to emotional distress and anxiety (i.e., a group high in emotional urgency and enhanced response to punishment).

Based on work examining patterns of psychopathology in emerging adolescence (49), we predicted that CD+ youth would exhibit high levels of both a general liability for broad psychopathology and variance specific to externalizing disorders. We also predicted that these indicators would differ as a function of personality profile, specifically with individuals with a high distress personality profile also scoring high on a broad liability for psychopathology, while a group marked by high levels of CU traits would display greater externalizing psychopathology. Given the exploratory nature of these analyses we did not have strong *a priori* hypotheses regarding differences in social, environmental, and neurocognitive factors by profiles.

## Methods

### Participants

The present study used data collected from youth and parents from the ABCD study, a large-scale study of youth aged 9–10 years (*N* = 11,875), recruited from 21 research sites across the United States (50–52), collected from baseline visits between September 1, 2016 and November 15, 2018 (ABCD Release 2.01, doi: 10.15154/1504041) We removed 323 participants from the analyses owing to non-response on the Kiddie-Structured Assessment for Affective Disorders and Schizophrenia for DSM-5 (KSAD-COMP), which was used to determine CD diagnosis, or grouping variables (Urgency, Premeditation, Perseverance, Sensation-seeking-Positive urgency [UPPS-P], Behavioral Inhibition System/Behavioral Activation System [BIS/BAS], CU traits), leaving a final sample size of 11,552 individuals, with 365 youth meeting criteria for CD. The participants with available data did not differ significantly from the full sample on key demographic variables (sex, race/ethnicity, household income, parental education, and marital status). A single IRB is maintained by the University of California San Diego Human Research Protections Program (160091). All parents provided written informed consent and all children provided assent.

The sample of 11,552 was roughly gender balanced (45.3% female) with a mean age of 9.92 years (*SD* = 0.62 years). Around half (49.6%) of the sample was white, with the remaining participants identifying themselves as Hispanic (19.0%), Black (14.2%), or Other/Multi-racial (10.4%). Approximately two-thirds of youth (64%) came from households in which the parents were married. Most parents (85.7%) reported an education level of at least some college, and most households reported an annual income of at least $50,000 (61.3%).

### Measures

Measures used in the study are described briefly below; however, please refer to the following papers for a more detailed description of the measures used in this study and their psychometric performance in the ABCD sample: personality and mental health (50); culture and environment (53); neurocognition (54).

#### Selection Variable

The KSAD-COMP is a structured, diagnostic interview that was administered to parents via computer in reference to their child (55). Parents completed all modules of the KSAD-COMP with the exception of the enuresis, encopresis, and selective mutism modules (50). For the purposes of the current study, participants were placed in the CD-positive (CD+) group if they met past or present DSM-5 criteria (e.g., at least three symptoms endorsed in the past 12 months, with at least one criterion present in the past 6 months) for CD at the time of the baseline assessment (age 9–10; e.g., childhood onset; *n* = 365).

#### Grouping Variables

A 20-item youth short-version of the *UPPS-P scale*, developed for use in the ABCD study (50), was administered via self-report at baseline to index trait impulsivity. The UPPS-P has five subscales: Negative Urgency, Positive Urgency, Lack of Perseverance, Lack of Planning, and Sensation Seeking.

The *BIS/BAS* measure (50, 56) is a 24-item youth-report scale designed to assess three facets of behavioral activation (BAS): Drive (intensity of goal directed behavior), Fun Seeking (spontaneity), and Reward Responsiveness (excitement over reinforcing outcomes), as well as a Behavioral Inhibition (BIS) scale, comprised of items tapping worry and fearfulness, with scores related to sensitivity to punishment as well as avoidance motivation.

A four-item youth-report measure of *CU traits* was developed to index lack of empathic concern, shallow affect, and low moral regulation within the ABCD study (57). This measure of CU traits was derived from three items (reversed) from the Strengths and Difficulties Questionnaire [SDQ; (58)] and one item from the Child Behavior Checklist [CBCL; (59)]. Scores were computed using a traditional summed score approach. This brief scale demonstrated adequate convergent and discriminant validity both in a subsample of the ABCD study as well as in separate validation samples (57).

#### Criterion Variables: Family History

*Family history of substance problems* was computed from ABCD's Family History Assessment (50). A threshold was established for a family member counting as an “affected case” based on the number of serious problems that person has had due to alcohol use and substance use. The following coding was used: 0 = neither parent met the threshold; 1 = at least one parent met threshold.

*Family history of psychopathology* was computed based on previously published protocols (60, 61). A family history composite score was constructed from responses for ABCD's Family History Assessment (50). Responses for the eight categories of psychopathology problems (e.g., depression, mania, ever psychiatrically hospitalized) were tabulated separately for first-degree relatives (mother, father, full siblings) and second-degree relatives (i.e., everyone else including aunts and uncles, grandparents, etc.). A weighted sum score was computed as follows: first degree cases + 0.5 ^*^ second degree cases. This number was then divided by the total possible score for the given subject, given the relations present in their own family tree. These proportion scores were calculated for each category (e.g., depression) and then summed across categories.

#### Criterion Variables: Dimensional Measures of Psychopathology

The *CBCL* [age 6–18 form; (59)] is a 119-item parent-report on child psychopathology. A general psychopathology [P-factor; (62, 63)] and orthogonal externalizing (EXT) and internalizing (INT) factors were modeled, and factor scores were used in subsequent analyses. Factor scores were derived by fitting a bifactor model to 8 CBCL scales [Withdrawn, Somatic Complaints, Anxious/Depressed, Social Problems, Thought Problems, Attention Problems, Delinquent Behavior, and Aggressive Behavior; (64)]. In this model there was a general P factor that all scales loaded onto (average scale loading on *P* = 0.69), and two specific factors: EXT and INT (average scale loading on sub-factors = 0.43). The EXT specific factor included the Delinquent and Aggressive Behaviors scales, while the INT specific factor included the Withdrawn, Somatic Complaints, and Anxious/Depressed scales. This model fit well-based on conventional fit thresholds (χ^2^ = 747.73, df = 16, *p* < 0.001; RMSEA = 0.062; CFI = 0.985; TLI = 0.974; SRMR = 0.015) and was chosen for its good model fit and theoretical interpretability (64). For additional information regarding modeling please refer to Clark et al. (64). Associations with the traditional CBCL scales were also included for comparison (see Supplementary Table 1).

#### Criterion Variables: Social Environment

Youth reported on *School Risk and Protective Factors (SRPF)* to assess their connection to the school environment (53). This 12-item measure was derived from the PhenX Toolkit, yielding three subscales: a six-item School Environment scale, a four-item School Involvement scale, and a two-item School Disengagement scale.

Both parents and children reported on *neighborhood safety and crime*, with items from the PhenX Toolkit (53). Parents responded to three statements regarding feelings of safety and presence of crime in their neighborhood, rated on a five-point Likert scale. Youth responded to only one item (“My neighborhood is safe from crime”), rated on the same 5-point Likert scale.

#### Criterion Variables: Social Functioning

Parent and child both rated the quality of family environment with the Family Conflict subscale from the *Family Environment Scale* (53). This 9-item measure from the PhenX Toolkit has a binary (true/false) response format to items related to presence of family conflict.

Youth reported on their perceived level of parental monitoring via the *Parental Monitoring Survey*, a five-item scale that was developed to assess parents efforts to keep track of their child's whereabouts at home as well as outside of the home (53).

Parents reported on their child's prosocial behavior using the *Prosocial Behavior Scale*, which is a three-item scale formed from the SDQ (65). Youth also responded; however, these three items were used in part to compute the 4-item CU Traits scale and were therefore not evaluated as a criterion variable.

#### Criterion Variables: Neurocognition

*General Cognitive Ability* (GCA) scores were computed by fitting a bifactor model to behavioral tasks from the NIH toolbox, the Rey Auditory Verbal Learning Task, the WISC-V, and the “Little Man” task (66). GCA factor scores were generated using expected a posteriori scoring (67).

### Data Analyses

We first examined whether youth with (*n* = 365) vs. without (*n* = 11,187) a CD diagnosis differed in demographic characteristics, personality traits, presence of comorbid psychopathology, or social environment characteristics. These analyses were conducted in SPSS (Version 25) and analyzed using data nested by site and family. Given the large sample size used for these analyses, effect sizes are also reported alongside the use of a strict significance threshold (*p* < 0.005) to increase interpretability of results. Then, in Mplus [version 8.4; (68)], we used LPA to identify mutually exclusive, latent subgroups of youth. Each of the 10 grouping variables (i.e., Positive Urgency, Negative Urgency, Lack of Planning, Lack of Perseverance, Sensation Seeking, BIS, BAS-Reward Responsivity, BAS-Drive, BAS-Fun Seeking, and CU traits) were input as profile indicators. We used the two-level, complex mixture analysis type in Mplus in order to account for clustering by study site and family identifiers. We tested six different LPA models using a step-wise approach, beginning with a one-profile model (see **Table 4**). We used empirical indicators to inform our selection of the best fitting model: (1) lower Akaike's Information Criterion (AIC)/Bayesian Information Criterion (BIC) values; (2) entropy closer to 1.00; and (3) non-significant Lo-Mendell-Rubin test. We then used class interpretability to determine the final model. As described in the literature, additional classes may show an improvement via empirical indicators but are not meaningful in relation to observable group differences (69).

Follow-up analyses examining latent profile differences on categorical covariates (i.e., demographics) were performed using the R3STEP auxiliary command in Mplus (70), which corrects for classification errors and provides model stability. The results of the R3STEP auxiliary command are provided through logistic regression models with each possible profile by profile comparison used as the outcome variable (i.e., Profile 1 vs. Profile 2, etc.). The Bolck, Croon, and Hagenaars (BCH) method was used to determine if there were latent profile differences on continuous outcome variables (70). The BCH method allows for the examination of continuous outcome variables while accounting for unequal variance among the outcome variables and measurement error of the latent profile using probabilities accounts (71). Given the relatively high number of omnibus and between-group analyses conducted, to minimize Type I error, the statistical significance threshold was *p* < 0.005.

## Results

### Characteristics of Youth With Childhood-Onset Conduct Disorder

Demographic characteristics for youth with (CD+) and without (CD–) a CD diagnosis are presented in Table 1. As expected, these groups differed in composition by sex (φ = −0.07), race/ethnicity (Cramer's *V* = 0.08), family income (*V* = 0.07), parental marital status (*V* = 0.08), highest parental education (*V* = 0.07), and family history of substance problems (φ = 0.10). The CD+ group had a higher proportion of males and Black and other/multiracial youth and were more likely to: (1) come from a lower income household, (2) have an unmarried parent, (3) have a lower rate of parents with at least a bachelor's degree, and (4) have a higher incidence rate of at least one parent having a substance use problem. The two groups did not differ significantly based on family history of psychopathology.

**Table 1:** Demographic descriptives.

	**CD–**	**CD+**	***X^**2**^ (df)***	***p***
	***n* = 11,187**	***n* = 365**		
	***n* (%)/** **M (SD)**	***n* (%)/** **M (SD)**		
**Sex**			**51.41 (1)**	**<0.001**
Female	5,409 (48%)	107 (29%)		
**Race/Ethnicity**			**68.06 (3)**	**<0.001**
White	5,891 (53%)	150 (41%)		
Black	1,622 (14%)	103 (28%)		
Hispanic	2,264 (20%)	52 (14%)		
Other/Multi-racial	1,117 (10%)	55 (15%)		
**Family Income**			**55.54 (2)**	**<0.001**
< $50,000	2,954 (26%)	159 (44%)		
$50,000–$100,000	2,913 (26%)	85 (23%)		
≥$100,000	4,378 (39%)	89 (24%)		
**Parental Marital Status**			**73.41 (1)**	**<0.001**
Married	7,628 (68%)	171 (47%)		
**Highest Parent Education**			**58.03 (4)**	**<0.001**
Some HS	529 (5%)	35 (10%)		
HS degree/GED	1,033 (9%)	53 (15%)		
Some college	2,865 (26%)	124 (34%)		
Bachelor's degree	2,863 (26%)	72 (20%)		
Masters or Professional Degree	3,883 (35%)	81 (22%)		
**FH Substance Problems**			**101.80 (1)**	**<0.001**
≥ 1 parent with substance problem	2,066 (18%)	141 (39%)		
**FH Psychopathology**	0.38 (0.47)	0.69 (0.63)	0.33 (1)	0.563

*Chi-square tests performed on data nested by site and family. CD–, youth without a conduct disorder diagnosis; CD+, youth with a conduct disorder diagnosis; FH, Family History; HS, High School*.

Regarding personality variables (Table 2), the CD+ group had significantly higher scores on all of the UPPS subscales as hypothesized (Cohen's *d*'s ranged from 0.41 for Negative Urgency to 0.15 for Sensation Seeking). The CD+ and CD- groups did not differ on BIS or on the BAS-Reward Response subscale; however, the CD+ group did score significantly higher on the BAS-Drive (*d* = 0.36), BAS-Fun Seeking (*d* = 0.37), and CU Traits (*d* = 0.73) scales. The CD+ group also scored higher on levels of broad psychopathology (*d* = 1.82) and EXT (*d* = 1.96) and there was a trend toward lower scores on the INT subfactor (*d* = −0.22; *p* = 0.009) in the CD+ group (Table 3). Youth in the CD+ group reported significantly lower scores on the School Environment scale (*d* = −0.22; Table 3), reflecting less educational resources, as well as significantly higher on the School Disengagement scale (*d* = 0.20). Both youth (*d* = −0.33) and parents (*d* = −0.27) in the CD+ group reported living in significantly less safe neighborhoods and had higher levels of family conflict (*d* youth= 0.40; *d* parent = 0.84). Youth in the CD+ group also reported having less parental monitoring (*d* = −0.26), and their parents reported they exhibited less prosocial behavior in comparison to youth in the CD– group (*d* = −1.05). Lastly, regarding neurocognitive functioning, youth in the CD+ group scored significantly lower on the GCA index (*d* = −0.46).

**Table 2:** Profile indicator variables.

	**CD–**	**CD+**	***Wald***	***p***	**Profile 1**	**Profile 2**	**Profile 3**	***Wald***	***p***
	***n* = 11,187**	***n* = 365**	***X^**2**^ (1)***		***n* = 131**	***n* = 190**	***n* = 44**	***X^**2**^ (2)***	
	**M (SD)**	**M (SD)**			**M (SD)**	**M (SD)**	**M (SD)**		
**UPPS**									
Positive urgency	7.96 (2.95)	8.85 (3.22)	26.86	<0.001	7.31 (2.67)^a, b^	9.21 (2.97)^a, c^	11.86 (3.25)^b, c^	83.96	<0.001
Negative urgency	8.46 (2.63)	9.58 (2.91)	51.33	<0.001	7.92 (2.40)^a, b^	10.30 (2.66)^a^	11.36 (3.06)^b^	90.36	<0.001
Lack of planning	7.72 (2.36)	8.37 (2.80)	18.82	<0.001	8.19 (2.32)^a, b^	7.44 (2.20)^a, c^	12.91 (12.00)^b, c^	249.82	<0.001
Lack of perseverance	7.03 (2.25)	7.46 (2.42)	11.26	0.001	7.77 (2.26)^a, b^	6.58 (1.78)^a, c^	10.32 (2.86)^b, c^	84.97	<0.001
Sensation seeking	9.76 (2.67)	10.17 (2.83)	7.78	0.005	9.24 (2.52)^a, b^	10.51 (2.91)^a^	11.50 (2.56)^b^	32.13	<0.001
**BIS/BAS**									
BIS	9.51 (3.74)	9.55 (4.10)	0.04	0.839	7.56 (3.25)^a^	10.98 (3.88)^a^	9.34 (4.80)	70.44	<0.001
Bas- reward response	11.00 (2.91)	11.36 (3.18)	4.89	0.027	8.70 (2.93)^a, b^	13.00 (2.03)^a^	12.23 (2.73)^b^	212.20	<0.001
Bas- drive	4.10 (3.04)	5.24 (3.41)	38.80	<0.001	2.65 (2.18)^a, b^	6.43 (3.01)^a^	7.80 (3.26)^b^	227.01	<0.001
Bas- fun seeking	5.67 (2.63)	6.66 (2.97)	39.19	<0.001	4.04 (2.02)^a, b^	8.01 (2.22)^a^	8.66 (2.71)^b^	312.45	<0.001
**CU Traits**	1.09 (1.19)	1.99 (1.45)	135.45	<0.001	2.18 (1.49)^a, b^	1.57 (1.10)^a, c^	3.25 (1.79)^b, c^	46.02	<0.001

*Raw mean values reported. Comparisons between CD+ and CD– groups determined from separate general linear models nested by site and family. Comparisons between latent profiles performed using Bolck, Croon, and Hagenaars (BCH) procedure. M, mean; SD, standard deviation; BIS/BAS, behavioral inhibition system/behavioral activation system; CU, callous-unemotional; CD–, youth without a conduct disorder diagnosis; CD+, youth with a conduct disorder diagnosis. Paired superscripts (e.g., a's) denotes significant group differences at p < 0.005*.

**Table 3:** Psychopathology, social functioning, and neurocognition.

	**CD–**	**CD+**	***Wald***	***p***	**Profile 1**	**Profile 2**	**Profile 3**	***Wald***	***p***
	***n* = 11,187**	***n* = 365**	***X^**2**^ (1)***		***n* = 131**	***n* = 190**	***n* = 44**	***X^**2**^ (2)***	
	**M (SD)**	**M (SD)**			**M (SD)**	**M (SD)**	**M (SD)**		
**CBCL Factor Scores**									
P	−0.06 (0.87)	1.66 (1.41)	545.81	<0.001	1.39 (1.40)^a^	1.70 (1.31)	2.34 (1.60)^a^	12.47	0.002
INT	0.00 (0.69)	−0.15 (1.13)	6.89	0.009	−0.03 (1.07)	−0.25 (1.17)	−0.11 (1.05)	3.22	0.200
EXT	−0.05 (0.72)	1.46 (1.56)	325.25	<0.001	1.11 (1.43)^a, b^	1.61 (1.59)^a^	1.90 (1.63)^b^	12.47	0.002
**School Risk and Protective Factors**									
School environment	19.95 (2.80)	19.31 (3.40)	225.69	<0.001	18.76 (3.44)^a^	19.94 (3.29)^a, b^	18.23 (3.28)^b^	15.98	<0.001
School involvement	13.06 (2.36)	12.81 (2.60)	3.04	0.081	12.46 (2.69)^a^	13.31 (2.35)^a, b^	11.75 (2.89)^b^	16.83	<0.001
School disengagement	3.74 (1.45)	4.04 (1.64)	12.12	<0.001	4.05 (1.64)	3.89 (1.65)^a^	4.61 (1.48)^a^	8.17	0.017
**Neighborhood Safety**									
Youth report	4.04 (1.08)	3.66 (1.31)	29.67	<0.001	3.78 (1.21)	3.59 (1.36)	3.57 (1.39)	1.85	0.397
Parent report	3.90 (0.97)	3.61 (1.11)	23.96	<0.001	3.78 (1.02)	3.50 (1.17)	3.60 (1.03)	5.59	0.061
**Family Conflict**									
Youth report	2.01 (1.94)	2.82 (2.23)	45.98	<0.001	2.42 (2.20)^a^	2.82 (2.09)^b^	3.98 (2.43)^a, b^	14.82	0.001
Parent report	2.47 (1.92)	4.31 (2.29)	225.69	<0.001	4.09 (2.17)	4.57 (2.32)	3.82 (2.41)	5.49	0.064
**Parental Monitoring**	4.39 (0.51)	4.25 (0.62)	17.22	<0.001	4.30 (0.57)	4.26 (0.64)	4.07 (0.66)	4.34	0.114
**Prosocial Behavior**									
Parent report	1.74 (0.39)	1.33 (0.55)	218.34	<0.001	1.32 (0.53)	1.36 (0.55)	1.25 (0.60)	1.44	0.487
**GCA**	0.08 (0.85)	−0.33 (0.91)	71.11	<0.001	−0.33 (0.92)	−0.47 (0.94)	−0.41 (1.06)	1.74	0.418

*Raw mean scores reported. Comparisons between CD+ and CD– groups determined from separate general linear model nested by site and family. Comparisons between latent profiles performed using Bolck, Croon, and Hagenaars (BCH) procedure. M, mean; SD, standard deviation; CD–, youth without a conduct disorder diagnosis; CD+, youth with a conduct disorder diagnosis; GCA, general cognitive ability. Paired superscripts (e.g., a's) denotes significant group differences at p < 0.005*.

### Trait Profiles of Youth With Childhood-Onset Conduct Disorder

Next, different patterns of trait impulsivity, inhibition, behavioral activation, and callousness were examined to account for heterogeneity within the CD+ group. Model fit and entropy statistics are presented in Table 4. The three-profile solution provided the best classification certainty via the highest entropy, and while the AIC and BIC improved slightly when defining a four-profile solution, the Lo-Mendell-Rubin test provided evidence that there was not a significant improvement in moving from a three- to four-profile model. Models were tested up to and including a six-profile model (Table 4); however, the three-profile solution was deemed to be substantively interpretable and therefore was retained for further analyses.

**Table 4:** Latent profile model fit statistics.

	**Profile proportions from posterior probabilities**	**AIC**	**Sample-size adjusted BIC**	**Lo-Mendell-Rubin significance**	**Entropy**
1 Profile	Profile 1: 100%	11155.14	11169.68		1.00
2 Profile	Profile 1: 42.80% Profile 2: 57.20%	10829.93	10852.47	1 v 2 *p* < 0.001	0.73
**3 Profile**	**Profile 1: 36.07%** **Profile 2: 51.33%** **Profile 3: 12.59%**	**10696.79**	**10727.33**	**2 v 3** ***p*** **= 0.10**	**0.78**
4 Profile	Profile 1: 18.53% Profile 2: 22.21% Profile 3: 46.09% Profile 4: 13.17%	10623.08	10661.62	3 v 4 *p* = 0.12	0.76
5 Profile	Profile 1: 19.51% Profile 2: 17.07% Profile 3: 35.24% Profile 4: 17.71% Profile 5: 10.42%	10567.47	10614.01	4 v 5 *p* = 0.20	0.75
6 Profile	Profile 1: 19.14% Profile 2: 16.95% Profile 3: 35.52% Profile 4: 16.27% Profile 5: 3.66% Profile 6: 8.46%	10518.72	10573.26	5 v 6 *p* = 0.43	0.79

*AIC, Akaike information criterion; BIC, Bayesian information criterion. Bolded value chosen as best fitting model*.

**Figure 1:**
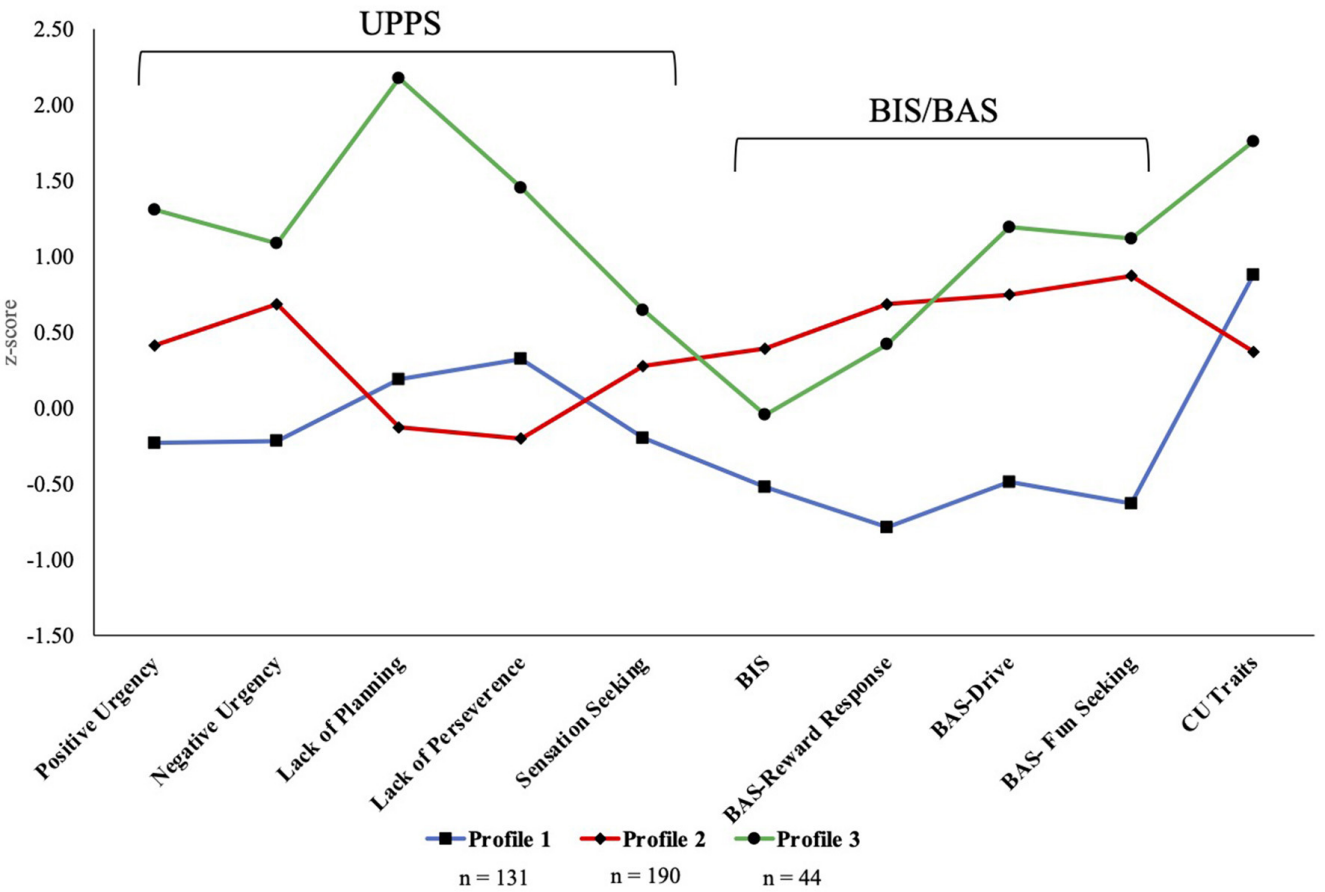
Personality type profiles. BIS, behavioral inhibition system; BAS, behavioral activation system; CU, callous unemotional.

The trait profile of the three-profile solution is depicted in Figure 1, and the trait values and results of difference tests are presented in Table 2. Profile 1 (*n* = 131) had the lowest scores on the Negative Urgency, Positive Urgency, Sensation Seeking, BIS, and BAS scales, and scored moderately high on Lack of Planning, Lack of Perseverance, and CU traits, defining a low impulsivity/BAS group. Profile 2 (*n* = 190) scored moderately high on Negative Urgency, Positive Urgency, BIS, and BAS scales, and lowest on Lack of Planning, Lack of Perseverance, and CU traits, defining a high Urgency/BAS group. Lastly, Profile 3 (*n* = 44) had the highest scores on all UPPS subscales, excluding Sensation Seeking, did not differ significantly from Profile 2 on BIS/BAS scale scores, and had the highest score on CU traits, defining a global high severity group.

### Characteristics of Trait Profiles

**Figure 2:**
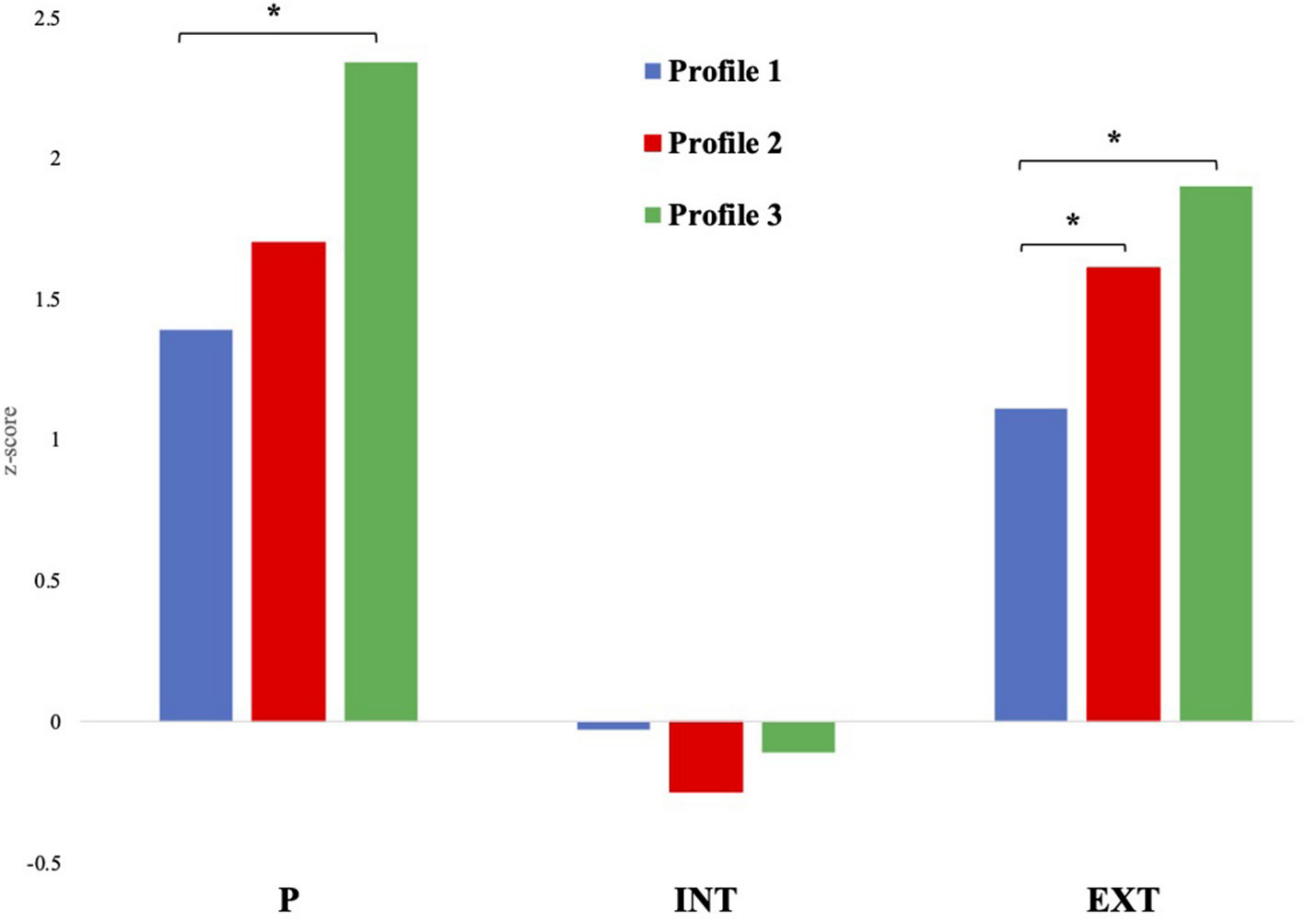
Latent profiles by General Factor of Psychopathology (P), Internalizing (INT), and Externalizing (EXT) factor scores from the Parent Report CBCL. *denotes differences in P, INT, and EXT factor scores that are significantly different at *p* < 0.005.

Demographic characteristics for the trait profiles are presented in Table 5. Youth in Profile 3 had significantly lower family income than youth in Profiles 1 and 2 (Odds Ratios = 0.52 and 0.44 respectively; Table 5). No other significant differences between profiles were observed for demographic variables. Profile differences in psychopathology, social environment, and neurocognition are presented in Table 3. Youth in Profile 3 (global high severity) were significantly higher than those in Profile 1 (low impulsivity/BAS) on P and EXT factor scores, while Profile 2 (high Urgency/BAS) was significantly higher than Profile 1 on EXT scores only (Table 3, Figure 2). The groups did not differ significantly on INT factor scores. Youth in Profile 2 reported significantly better school environment and higher rates of school involvement, and the lowest rates of school disengagement. The youth in Profile 3 reported rates of family conflict that were significantly higher than individuals in Profile 1 and 2; however, this finding was not consistent in the parent report of family conflict, where none of the profiles differed significantly. The profiles did not differ significantly on level of parental monitoring or parent report of prosocial behavior. Regarding neurocognition, the three profile types also did not differ significantly from each other.

**Table 5:** Comparisons of latent profiles on demographic variables.

	**OR**	***p***	**OR**	***p***	**OR**	***p***
	**(95% CI)**		**(95% CI)**		**(95% CI)**	
	**1 vs. 2**		**1 vs. 3**		**2 vs. 3**	
Sex	1.08 (0.44, 2.41)	0.396	1.32 (0.07, 2.57)	0.617	0.93 (0.09, 1.76)	0.861
Race/ethnicity	1.26 (0.93, 1.59)	0.122	1.10 (0.73, 1.46)	0.611	0.87 (0.62, 1.12)	0.309
Family income	1.19 (0.51, 1.86)	0.590	0.52 (0.15, 0.89)	0.012	0.44 (0.17, 0.71)	<0.001
Parental marital status	0.67 (0.15, 1.19)	0.208	1.03 (0.04, 2.01)	0.959	1.53 (0.13, 2.93)	0.457
Highest parent education	0.82 (0.53, 1.10)	0.211	1.30 (0.71, 1.89)	0.321	1.59 (0.95, 2.23)	0.072
FH substance problems	1.51 (0.65, 2.37)	0.247	1.47 (0.34, 2.60)	0.414	0.97 (0.23, 1.72)	0.945
	***X**^**2**^**(1)***	***p***	***X**^**2**^**(1)***	***p***	***X**^**2**^**(1)***	***p***
	**1 vs. 2**		**1 vs. 3**		**2 vs. 3**	
FH psychopathology	2.78	0.096	1.34	0.247	0.02	0.883

*R3STEP and BCH analyses performed on data nested by site and family. OR, Odds Ratio; CI, Confidence Interval; FH, Family History. FH of psychopathology is a continuous variable and therefore the comparisons between latent profiles performed using Bolck, Croon, and Hagenaars (BCH) procedure. All other analyses used the R3STEP analytic approach to test for differences between latent profiles*.

## Discussion

The current study used person-centered analyses to characterize heterogeneity on multiple trait dimensions of youth who met criteria for childhood-onset CD. As expected, and consistent with previous studies on childhood-onset CD (1, 9, 48), youth who met criteria for CD were higher on trait measures of impulsivity, behavioral activation subscales, and CU traits. Of note, these youth did not differ from the CD- group on sensitivity to punishment (BIS) and the BAS-Reward Responsivity subscale. While these subscales proved informative for defining latent profiles, this heterogeneity is lost when examining differences between the CD+ vs. CD– youth. As scores on these subscales have been linked to differences in neural and psychophysiological response (31, 41, 42), these findings demonstrate a strength of the study: the characterization of meaningful groups that would not otherwise emerge when simply examining groups defined by the DSM-5 criteria. CD+ youth also scored significantly higher on an aggregate liability for broad psychopathology and externalizing psychopathology with a trend-level *lower* scores on liability for internalizing psychopathology, consistent with previous research on the nomological networks of the psychopathology factors (49). In addition, the CD+ youth had significant environmental, social, and neurocognitive impairments.

We were also able to define meaningful profiles based on differing personality trait scores within the CD+ group, further accounting for heterogeneity within this high-risk group. The LPA identified three personality profiles that were generally consistent with our hypotheses. For example, while Profiles 1 and 3 included youth who scored relatively high on CU traits, they differed on trait impulsivity, suggesting that although these two traits can co-occur, they represent distinct dimensions relevant to CD symptoms. In addition, the youth who scored lower on CU traits (Profile 2) also had the highest scores on BIS, reflecting a sensitivity to punishment that is thought to be reduced in individuals high on CU traits (33–37). Researchers have found mixed evidence for the association between CU traits and reward responsivity (12, 39). The profiles that emerged in the current study may reconcile this somewhat, as youth in Profiles 2 and 3 were relatively matched on BAS-Reward Response scores but differed significantly on level of CU traits. Furthermore, Profiles 1 and 3 significantly differed on emotional impulsivity, with one group scoring in a normative range on Negative and Positive Urgency (Profile 1) and the other group scoring approximately one standard deviation higher than the full sample (*N* = 11,552; Profile 3).

These profiles also defined groups that differed significantly on clinically relevant variables. The P factor is conceptualized as a broad liability for all forms of prevalent psychiatric symptomatology, while the EXT and INT factors represent more of a domain-specific liability (49, 72, 73). Therefore, different factor scores on P and EXT for the different profiles suggests that these latent profiles provide meaningful information regarding risk for psychopathology. For example, individuals in Profile 3 scored significantly higher on both the P factor and the EXT subfactor (in comparison to Profile 1) consistent with the characterization of a high global severity group. This suggests that these youth are exhibiting psychopathology beyond CD and may also be at risk for displaying this pattern of broad psychological distress as they develop. In comparison, youth in Profile 2 scored significantly higher only on EXT compared to Profile 1, suggesting that this personality profile is selectively associated with risk for externalizing, but not general psychopathology. Future work to better characterize the patterns of comorbidity of these profiles is needed to determine the stability of these associations, as well as to examine associations with psychopathology that typically has an onset in adolescence (i.e., substance use disorders), and therefore cannot be reliably studied at this age in a community sample.

The groups defined by the latent profiles also differed on some environmental and social variables. Youth in Profile 2 had significantly higher levels of school enrichment and involvement than those in Profiles 1 and 3 and were less disengaged in school compared to the youth in Profile 3. In addition, Profile 3 youth reported significantly higher levels of family conflict than those in Profiles 1 and 3. These findings are of interest as the youth in Profile 1 appear to be relatively low risk—they have the lowest scores on the P and EXT factors, are only elevated on CU traits, and score in a relatively normative range (within ±0.50 standard deviation of the full sample) on impulsivity and responsivity to punishment and reward. Further research is needed to determine the developmental trajectory of these youth as, based on the current cluster of risk/protective factors, we would hypothesize that CD symptoms in this group is likely to remit over time. Beyond these findings, these youth reported relatively similar scores on environmental variables such as neighborhood safety and parental monitoring. It appears that these variables instead may act as general contextual risk factors for CD, but not through pathways unique to the characteristics of one of the profiles defined by the LPA.

## Limitations

While the findings from the current study are novel, there are limitations that must be noted. One limitation of the present study is that of generalizability. While the ABCD study was recruited to be nationally representative based on demographic variables, this does not mean that these findings will generalize into other samples of interest including clinical populations. Additionally, the three subgroups can also be interpreted as being “severity” profiles within individuals that meet criteria for a CD diagnosis. A deeper understanding of how CD symptom severity relates to psychopathology, social functioning, and neurocognition has important implications for clinical practice. For example, the high severity group defined by these analyses (i.e., Profile 3) was also at highest risk for general psychopathology (i.e., the P factor) and therefore youth with severe CD symptoms should be assessed for mental health problems beyond externalizing disorders.

In addition, the study was limited by the measures available for use in the LPA. The ABCD study was not explicitly designed to answer this research question and therefore additional indicators [e.g., the Inventory of Callous Unemotional Traits, (74)] and criterion variables (e.g., detailed information about delinquency) were not available. In addition, alternative models that describe the emergence and persistence of antisocial behavior in youth emphasize the presence of grandiose-manipulative traits (75, 76) alongside CU and impulsive-irresponsible traits. Therefore, future work will be needed to determine if self-report of grandiose-manipulative traits provides additional differentiation of profiles and if these profiles replicate in datasets that have additional scales and criterion variables.

Lastly, the diagnostic instrument (KSAD-COMP) was completed by a parent and not a trained clinician. As symptoms of CD are highly behavioral and observable, parent-ratings are likely to be valid. In support of this, youth with CD diagnoses scored significantly higher on CBCL Externalizing Problems and the Conduct Problems scale (Supplementary Table 1) providing convergent evidence for the validity of the measure. Future waves of data collection of the ABCD study will include youth self-report of DSM-5 symptoms, which can be used for further validation of the profiles identified in this study.

## Conclusions and Future Directions

These results are valuable for understanding antisocial behavior in youth and how different configurations of personality traits can provide relevant information to further understand heterogeneity within the childhood-onset CD diagnosis. For example, youth within Profile 3 were elevated on CU trait as well as trait indices of distress (Negative/Positive Urgency) and behavioral activation. These youth might therefore benefit from treatments focused on regulating emotions, increasing prosocial behavior, and implementing behavioral interventions to increase planfulness. In comparison, the youth in Profile 1 also scored relatively high on CU traits and therefore would likely benefit from interventions to increase prosocial behavior but would not need additional interventions targeting distress and impulsivity.

In addition, these findings provide information regarding how these dimensions interact to form distinct profiles within youth diagnosed with childhood-onset CD. While impulsivity, punishment and reward responsivity, and CU traits have each been studied as unique risk factors for antisocial behavior, this is the first study to examine how these risk factors co-occur. As these systems rely on shared neural architecture [e.g., amygdala response is linked to both reward responsivity and CU traits; (17, 39, 77)], a nuanced understanding of how these traits co-occur may help to clarify our understanding of individual differences in neural response of youth who demonstrate early antisocial behavior. Future research with the goal of replicating these profiles in additional datasets and across development is needed to determine the generalizability and stability of these patterns. Although the data used in this study are cross-sectional, data collection in the ABCD study is ongoing, and these findings therefore set the stage for prospective longitudinal research using multimodal data—such as neuroimaging and parent- and teacher-reports—to more fully determine how these latent profiles account for the course, severity, and comorbidity of psychopathology in these groups of high-risk youth.

## Data Availability Statement

Publicly available datasets were analyzed in this study. This data can be found here: The ABCD data repository grows and changes over time. The ABCD data used in this report came from doi: 10.15154/1504041 DOIs can be found at http://dx.doi.org/10.15154/1504041.

## Ethics Statement

The studies involving human participants were reviewed and approved by The ABCD Study Coordinating Center (CC) has already established a single IRB to facilitate uniform ethical standards are applied across sites and to comply with the NIH Policy on the use of a single IRB for multi-site research. The UCSD Human Research Protections Program (HRPP) is acting as the single IRB for the ABCD-USA consortium. This has improved quality and efficiency in protection of research participants. The University of Michigan has entered into a reliance agreement with the UCSD IRB and has ceded review to the single IRB at UCSD. The UCSD single IRB maintains all documents relating to the reliance agreements with the relying IRBs. Written informed consent to participate in this study was provided by the participants' legal guardian/next of kin.

## Author Contributions

SB and MH developed the study concept and design. SB and MM performed the data analysis. All authors contributed to the interpretation of the results, drafted the paper, and provided critical revisions. All authors approved the final version of the paper for submission.

## Conflict of Interest

The authors declare that the research was conducted in the absence of any commercial or financial relationships that could be construed as a potential conflict of interest.
