# Supplementary material for: Heterogeneity Within Youth With Childhood-Onset Conduct Disorder in the ABCD Study
Source: Front Psychiatry. 2021 Jul 16;12:701199. doi: 10.3389/fpsyt.2021.701199 (PMC8322519; doi:10.3389/fpsyt.2021.701199)
Supplement: Supplementary file 1 [file Data_Sheet_1.docx]

Supplemental Materials

Supplemental Table 1. Reliabilities of self-report scales.

|  | ***N* items** | **Chronbach’s Alpha** |
| --- | --- | --- |
| **UPPS** |  |  |
| Positive Urgency | 4 | .77 |
| Negative Urgency | 4 | .63 |
| Lack of Planning | 4 | .73 |
| Lack of Perseverance | 4 | .70 |
| Sensation Seeking | 4 | .49 |
| **BIS/BAS** |  |  |
| BIS | 7 | .63 |
| BAS- Reward Response | 5 | .73 |
| BAS- Drive | 4 | .77 |
| BAS- Fun Seeking | 4 | .66 |
| **CU Traits** | 4 | .50 |
| **School Risk and Protective Factors** |  |  |
| School Environment | 6 | .61 |
| School Involvement | 4 | .65 |
| School Disengagement | 2 | .20 |
| **Neighborhood Safety** |  |  |
| Parent Report | 3 | .88 |
| **Family Conflict** |  |  |
| Youth Report | 9 | .68 |
| Parent Report | 9 | .64 |
| **Parental Monitoring** | 5 | .46 |
| **Prosocial Behavior** |  |  |
| Parent Report | 3 | .58 |

Supplemental Table 2. Parent Report of Psychopathology

|  | **CD-** | **CD+** | ***Wald*** | ***p*** | **Profile 1** | **Profile 2** | **Profile 3** | ***Wald*** | ***p*** |
| --- | --- | --- | --- | --- | --- | --- | --- | --- | --- |
|  | ***n* = 11,187** | ***n* = 365** | ***X^2^ (1)*** |  | ***n* = 131** | ***n* = 190** | ***n* = 44** | ***X^2^ (2)*** |  |
|  | **M (SD)** | **M (SD)** |  |  | **M (SD)** | **M (SD)** | **M (SD)** |  |  |
| **DSM5 Scales** |  |  |  |  |  |  |  |  |  |
| Depressive Problems | 53.40 (5.51) | 59.87 (8.30) | 220.72 | <.001 | 59.03 (8.46) | 59.83 (8.02) | 62.13 (8.98) | 3.97 | .137 |
| Anxiety Problems | 53.32 (5.96) | 58.32 (8.68) | 117.89 | <.001 | 57.86 (8.79) | 57.92 (8.08) | 61.24 (10.59) | 4.20 | .123 |
| Somatic Problems | 55.36 (6.56) | 58.84 (7.72) | 71.01 | <.001 | 57.11 (7.25) ^a^ | 59.02 (7.72) | 62.20 (7.78) ^a^ | 14.72 | .001 |
| ADH Problems | 52.96 (5.30) | 61.38 (8.80) | 330.69 | <.001 | 59.32 (8.64) ^a^ | 61.60 (8.43) | 65.38 (9.53) ^a^ | 14.61 | .001 |
| OD Problems | 53.16 (4.98) | 63.15 (8.37) | 506.34 | <.001 | 62.64 (8.28) | 62.69 (8.23) | 66.53 (8.42) | 8.43 | .015 |
| Conduct Problems | 52.56 (4.76) | 66.94 (8.16) | 1053.80 | <.001 | 65.15 (8.19) ^a^ | 67.17 (8.16) | 70.2 (6.96) ^a^ | 15.01 | .001 |

*Note*. Raw mean t-scored values reported. OC = Obsessive-Compulsive; ADH= Attention Deficit/ Hyperactivity; OD= Oppositional Defiant. Comparisons between CD+ and CD- groups determined from separate general linear models nested by site and family. Comparisons between latent profiles performed using Bolck, Croon, and Hagenaars (BCH) procedure. M = mean; SD= standard deviation; CD- = youth without a conduct disorder diagnosis; CD+ = youth with a conduct disorder diagnosis. Paired superscripts (e.g., a’s) denotes significant group differences at *p* < .005.

**Supplemental Figure 1.** Personality type profiles from 4-profile solution. BIS= behavioral inhibition system; BAS = Behavioral activation system; CU = callous-unemotional.
